# Anatomical Atlas, Illustrative of the Structure of the Human Body

**Published:** 1843-11-25

**Authors:** 


*Anatomical Atlas, Illustrative of the Structure of the Human Body.* By Henry H. Smith, M. D., etc. etc. Under the Supervision of William E. Horner, M. D., Professor of Anatomy in the University of Pennsylvania, etc. Part I. 130 Figures. Philadelphia: Lea & Blanchard. 1844. 8vo. pp. 56.

This is an exquisite volume, and a beautiful specimen of art. The utility of illustrations in acquiring a knowledge of a purely practical art, is universally admitted. The student of anatomy refers to his plates in his closet, in order to follow the description in the text book. The practitioner finds himself constantly obliged to refresh his memory on some anatomical point, and when circumstances do not admit of a direct appeal to the subject, drawings accomplish his purpose. We have numerous anatomical atlases, but we venture to say none equal in cheapness to the present volume, and none surpass it in faithfulness and spirit. Its claim, as is stated in the preface, is to have been selected from the most accurate sources, as well as from the latest microscopical observations on the anatomy of the tissues; and where plates were not deemed satisfactory, to have been enriched by original drawings, from specimens furnished by the Anatomical Museum of the University of Pennsylvania.

"In the arrangement of the work it will be seen that reference has been had to the production of a volume suited to general circulation, of such a size as could be conveniently used in the lecture, dissecting, and operating room, with a Terminology sanctioned by general usage in the United States, and with concomitant references on the same page, thereby saving to the young student much embarrassment and confusion. Lastly, it has been placed at such a price as will render it easy of acquirement by all."—*Preface.*

We again have much pleasure in referring to Mr. Gilbert as the artist; he has already acquired a deserved reputation in this branch of his art; and on this occasion he has been seconded by the printer, the cuts being most admirably "worked off,"—we believe that is the technical phrase.

We strongly reccommend to our friends, both urban and rural, who need anatomical plates, the purchase of this most excellent work, for which both editor and publishers deserve the thanks of the profession.

